# Super-pigment: Deciphering shikonin biosynthesis to fight cancer, inflammation, and much more

**DOI:** 10.1093/plphys/kiae210

**Published:** 2024-04-10

**Authors:** Lara Pereira

**Affiliations:** Assistant Features Editor, Plant Physiology, American Society of Plant Biologists; Ecology and Evolutionary Biology, School of Biosciences, University of Sheffield, Sheffield S10 2TN, UK

The roots of red gromwell (*Lithospermum erythrorhizon*) produce shikonin, a unique 1,4-naphthoquinone with a broad range of applications. Shikonin is a precious red pigment for the Asian culture, not only as a dye or cosmetic but also as an exceptional traditional herbal medicine. Shikonin has anti-inflammatory and antimicrobial properties and anticancer activity and helps with wound healing, among many other pharmacological uses (Yadav et al. 2022). For decades, shikonin and shikonin-derived metabolites have been produced industrially in cell cultures. However, despite its commercial value, the biosynthetic pathway of shikonin has not been completely solved.

Shikonin belongs to the broad metabolite class of phenylpropanoids. Other important and widely distributed phenylpropanoids are the ubiquitous cell wall component lignin and the antioxidants flavonoids. Also, all plants produce p-hydroxybenzoic acid (PHB) as a precursor of ubiquinone. Yet, shikonins are exclusively synthesized in a few species from the *Boraginaceae* family (Oshikiri et al. 2024).

In addition to shikonins, *L. erythrorhizon* produces the biosynthetically related compounds echinofurans and caffeic acid derivatives. Many of the steps in the biosynthetic pathway that produces these compounds are well-known, but the formation of the common precursors *p*-coumaroyl-CoA and PHB has been a long-standing question. The key enzyme 4-coumaroyl-CoA ligase (4CL) has been characterized in several plant species, but so far, no 4CL activity has been related to shikonin production.

In a research article of this issue of *Plant Physiology*, Nakanishi et al. (2024) investigate the role of 4CL in shikonin biosynthesis in *L. erythrorhizon* from a multi-disciplinary approach, including biochemical assays, transcriptomics, metabolite quantification, and reverse genetics. They concluded that two 4CL paralog genes, *Le4CL3* and *Le4CL4*, are involved in the biosynthesis of shikonins and echinofurans, and therefore these enzymes are likely needed to synthesize their common intermediate 3″-hydroxygeranylhydroquinone (Fig. 1).

**Figure 1.**
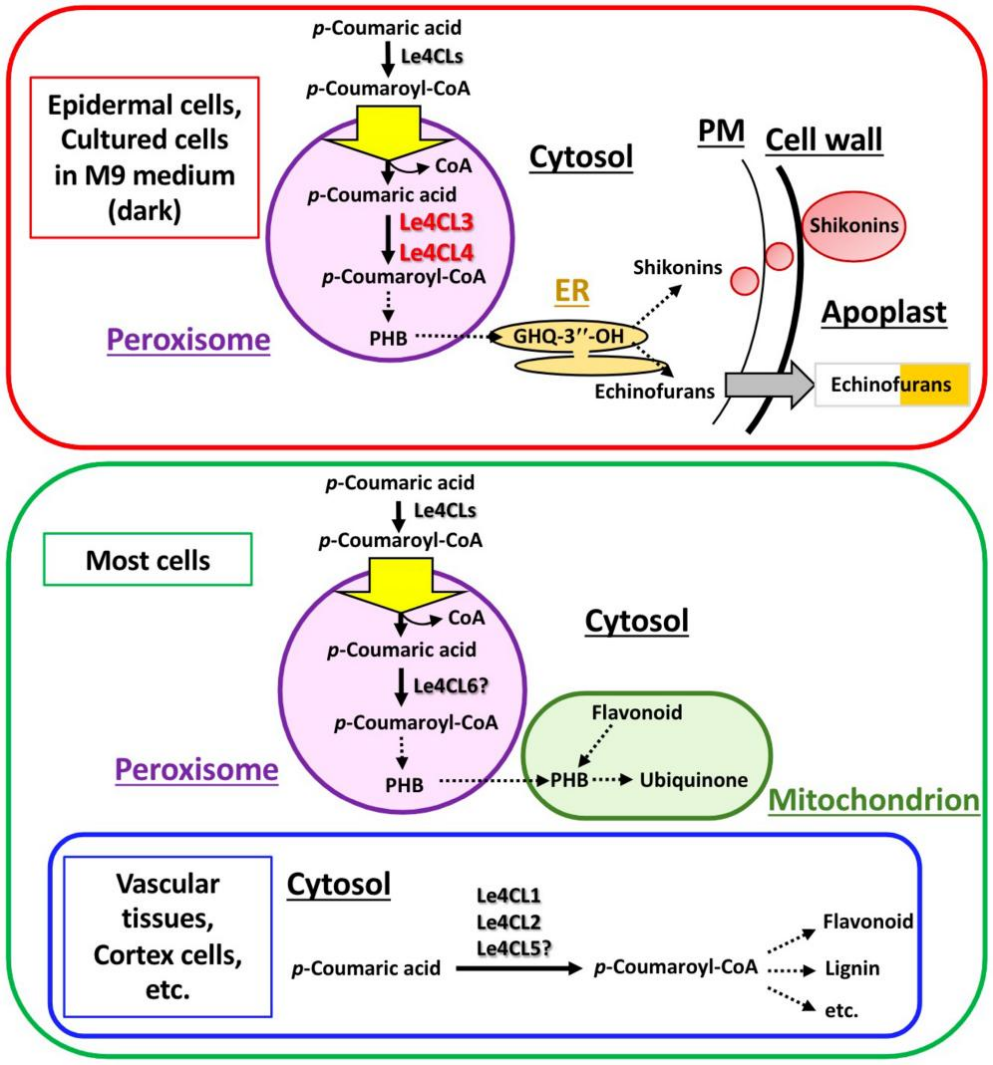
Functional specification of *Le4CL* paralogs in *L. erythrorhizon*. Peroxisome-localized Le4CL3 and Le4CL4 are responsible for the formation of PHB and subsequent prenylation in the endoplasmic reticulum (ER) for shikonin and echinofuran biosynthesis, which occurs in epidermal cells. In turn, Le4CL6 is thought to be involved in ubiquinone biosynthesis by providing PHB, while the further biosynthetic reactions take place in the mitochondria in most cells. Classical Le4CL paralogs such as Le4CL1, Le4CL2, and Le4CL5 may function in the cytosol or on the cytosolic side of the ER and are involved in the formation of other aromatic metabolites. Flavonoids are synthesized in cortex and other cells, and lignin is mainly synthesized in the vascular tissues of herbaceous plants. Arrows indicate enzymatic reaction steps and dashed arrows indicate multiple reaction steps. GHQ-3″-OH, 3″- hydroxygeranylhydroquinone; PM, plasma membrane [modified from Nakanishi et al. (2024)].

Initially, they cleverly used a 4CL inhibitor in *L. erythrorhizon* cultured cells to test whether shikonin production was altered and found out that shikonins and echinofurans levels were decreased. Then, the eight 4CL paralogs were narrowed down to 2, *Le4CL3* and *Le4CL4*, by selecting the genes that were preferentially expressed in roots under dark conditions and in shikonin-producing species. These 2 enzymes act in the peroxisome, in contrast with previously characterized 4CL1 and 4CL2 that are likely localized in the cytosol (Fig. 1).

To validate the function of these enzymes, CRISPR/Cas9 frameshift mutants were generated and the cells cultured in shikonin-producing media. Metabolite quantification by HPLC showed that indeed, shikonin and echinofuran production in the mutants was heavily reduced. In fact, the authors hypothesized that Le4CL3 and Le4CL4 might form a protein complex and may be unable to function when one of the proteins is absent, yet future research needs to be conducted to test this hypothesis.

Interestingly, *Le4CL3* and *Le4CL4* are single exon genes as well as previously characterized prenyltransferases also involved in shikonin biosynthesis in *L. erythrorhizon* (Kusano et al. 2019). The authors discussed that the reverse transcription of mature mRNA might be a possible explanation for the overrepresentation of single exon genes in specialized metabolism and how, if this observation can be generalized as a “molecular rule,” it would facilitate genome mining of specialized metabolism genes. Although this model may be too simplistic, since there are multiple genes involved in specialized metabolism in plants that contain introns, the idea of using sequence features to predict in silico biosynthetic genes is crucial, as it would certainly accelerate pathway discovery. Machine learning approaches that consider several sequence features, such as duplication pattern and sequence conservation, had been applied to propose genes involved in specialized metabolism (Moore et al. 2019).

Shikonin production in *L. erythrorhizon* cells has been employed for decades, and several factors, including light, media composition, and temperature, have been optimized to increase metabolite production (Yazaki 2017). Recently, the genome of *L. erythrorhizon* has been sequenced, offering a useful resource to further investigate the genetics behind shikonin production (Auber et al. 2020; Tang 2021). Yet many of the biosynthetic steps of these relevant metabolites are unknown, precluding the application of novel technologies and approaches, such as heterologous in vitro production by metabolic engineering and synthetic biology (García-Granados et al. 2019), from improving shikonin production. By characterizing a key enzyme in the pathway, Nakanishi et al. (2024) brought us closer to that final aim of an efficient, easy, and sustainable system to produce shikonin.

## References

[kiae210-B1] Auber RP , Suttiyut T, McCoy RM, Ghaste M, Crook JW, Pendleton AL, Widhalm JR, Wisecaver JH. Hybrid de novo genome assembly of red gromwell (Lithospermum erythrorhizon) reveals evolutionary insight into shikonin biosynthesis. Hortic Res. 2020:7(1):82. 10.1038/s41438-020-0301-9 32528694 PMC7261806

[kiae210-B2] García-Granados R , Lerma-Escalera JA, Morones-Ramírez JR. Metabolic engineering and synthetic biology: synergies, future, and challenges. Front Bioeng Biotechnol. 2019:7:36. 10.3389/fbioe.2019.00036 30886847 PMC6409320

[kiae210-B3] Kusano H , Li H, Minami H, Kato Y, Tabata H, Yazaki K. Evolutionary developments in plant specialized metabolism, exemplified by two transferase families. Front Plant Sci. 2019:10:794. 10.3389/fpls.2019.00794 31293605 PMC6603238

[kiae210-B4] Moore BM , Wang P, Fan P, Leong B, Schenck CA, Lloyd JP, Lehti-Shiu MD, Last RL, Pichersky E, Shiu S-H. Robust predictions of specialized metabolism genes through machine learning. Proc Natl Acad Sci U S A. 2019:116(6):2344–2353. 10.1073/pnas.1817074116 30674669 PMC6369796

[kiae210-B5] Nakanishi K , Li H, Ichino T, Tatsumi K, Osakabe K, Watanabe B, Shimomura K, Yazaki K. Peroxisomal 4-coumaroyl-CoA ligases participate in shikonin production in *Lithospermum erythrorhizon*. Plant Physiol. 2024:195(4):2843–2859. 10.1093/plphys/kiae157 38478427

[kiae210-B6] Oshikiri H , Li H, Manabe M, Yamamoto H, Yazaki K, Takanashi K. Comparative analysis of shikonin and alkannin acyltransferases reveals their functional conservation in Boraginaceae. Plant Cell Physiol. 2024:1–10. pcad158. 10.1093/pcp/pcad158. 38181221

[kiae210-B7] Tang C . Exploring the evolutionary process of alkannin/shikonin *O*- acyltransferases by a reliable *Lithospermum erythrorhizon* genome. DNA Res. 2021:28(5):dsab015. 10.1093/dnares/dsab015 PMC8435551 34424327

[kiae210-B8] Yadav S , Sharma A, Nayik GA, Cooper R, Bhardwaj G, Sohal HS, Mutreja V, Kaur R, Areche FO, AlOudat M, et al Review of shikonin and derivatives: isolation, chemistry, biosynthesis, pharmacology and toxicology. Front Pharmacol. 2022:13:905755. 10.3389/fphar.2022.905755 35847041 PMC9283906

[kiae210-B9] Yazaki K . *Lithospermum erythrorhizon* cell cultures: present and future aspects. Plant Biotechnol. 2017:34(3):131–142. 10.5511/plantbiotechnology.17.0823a PMC6565996 31275019

